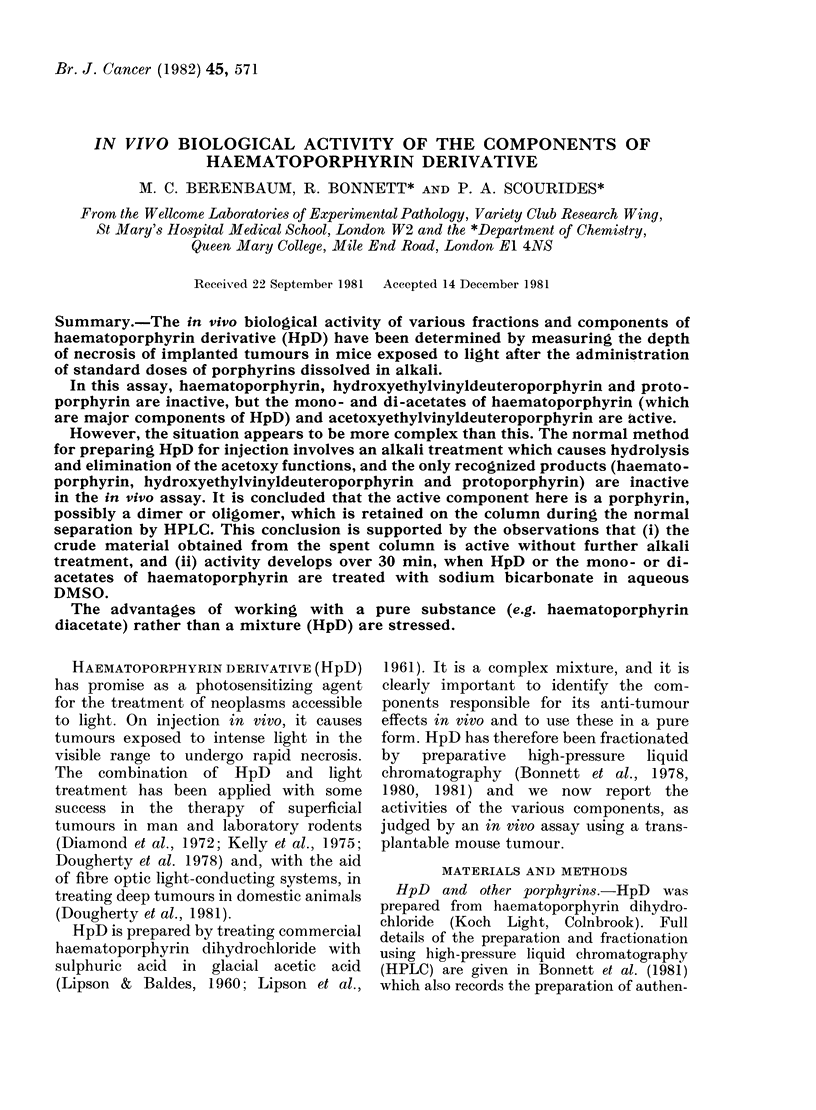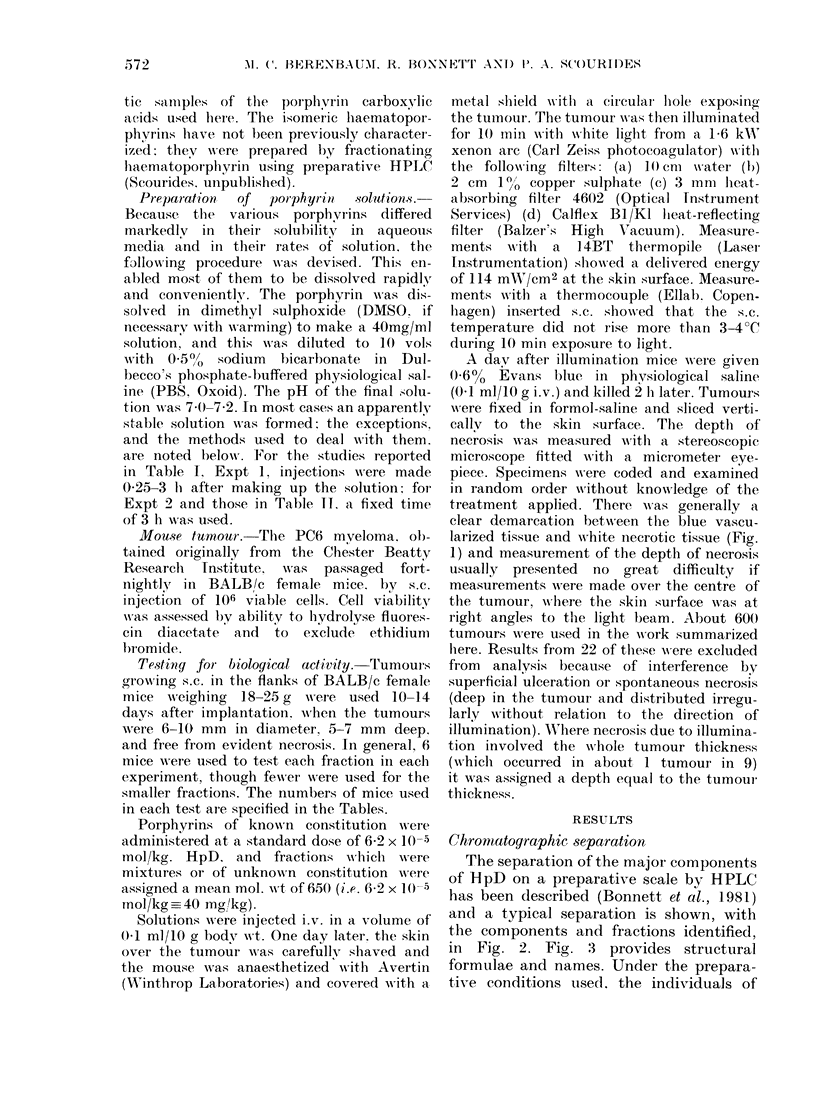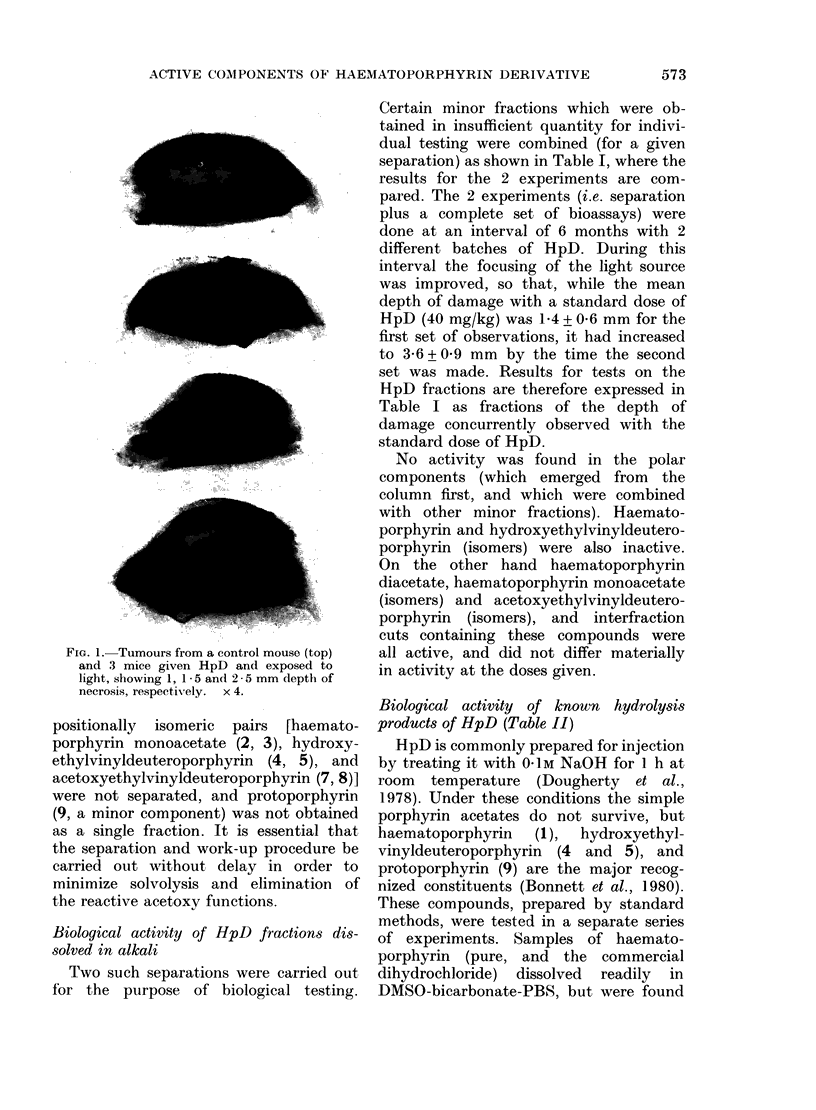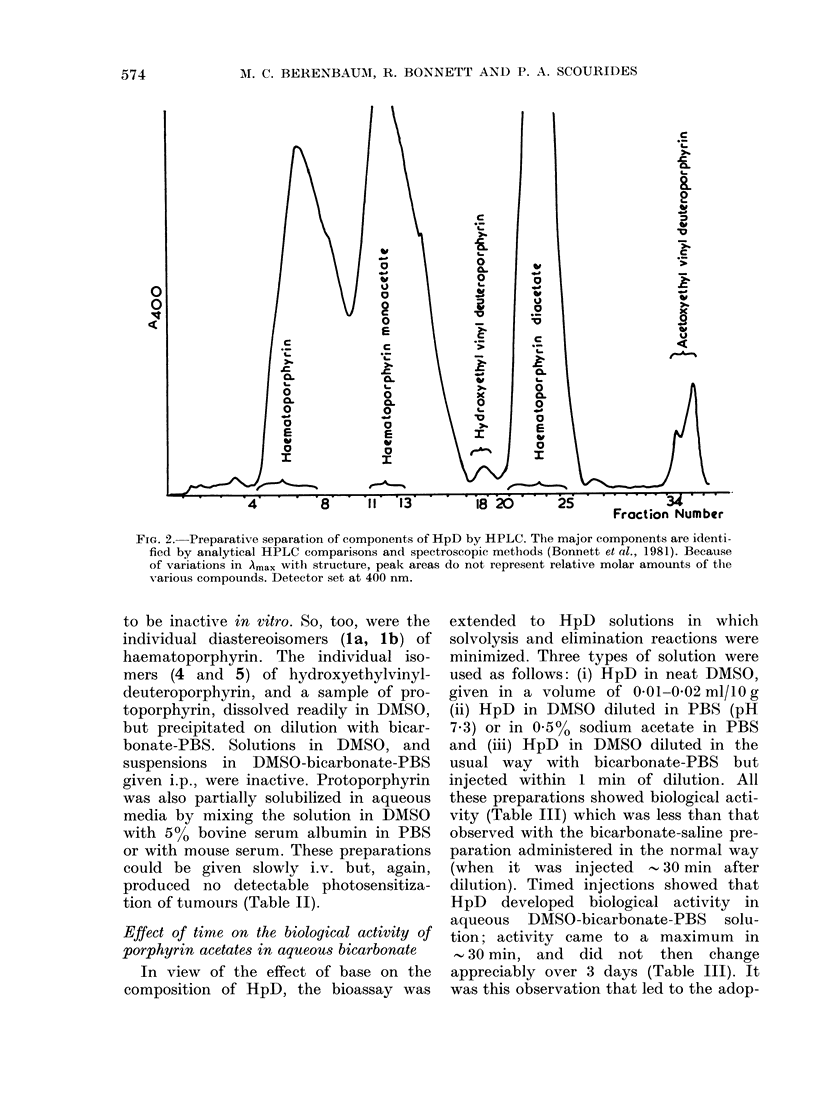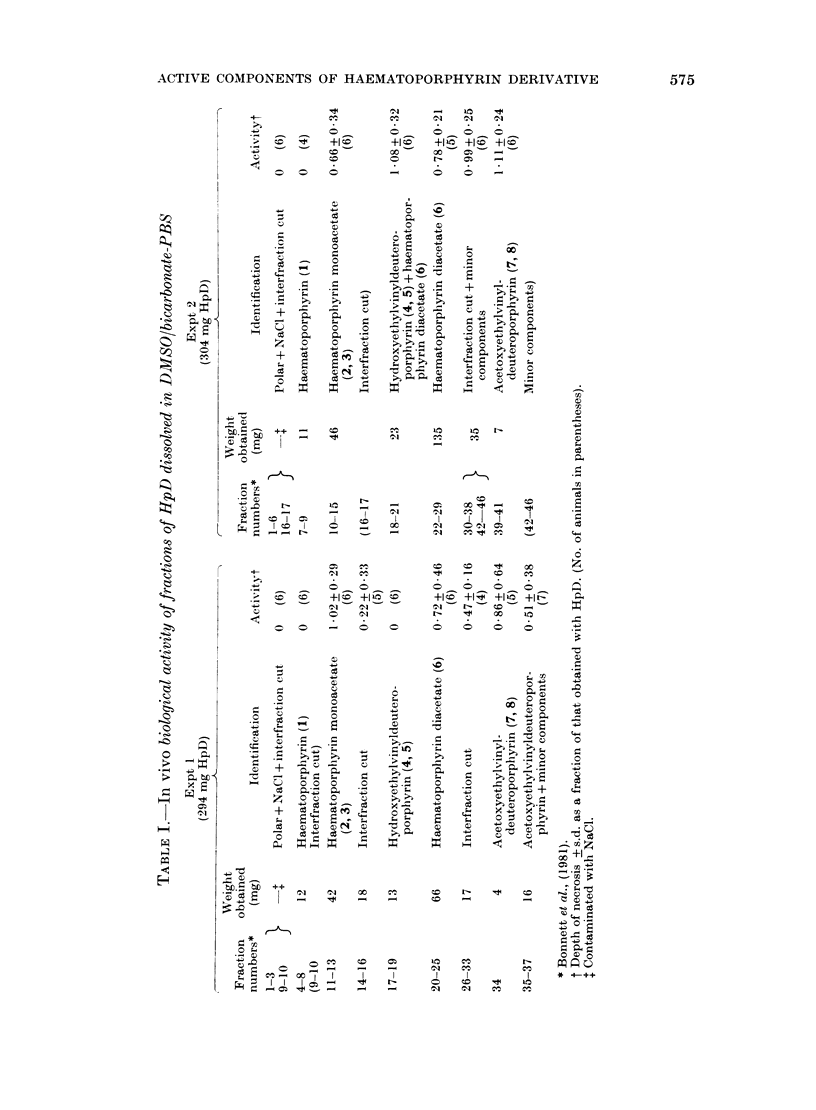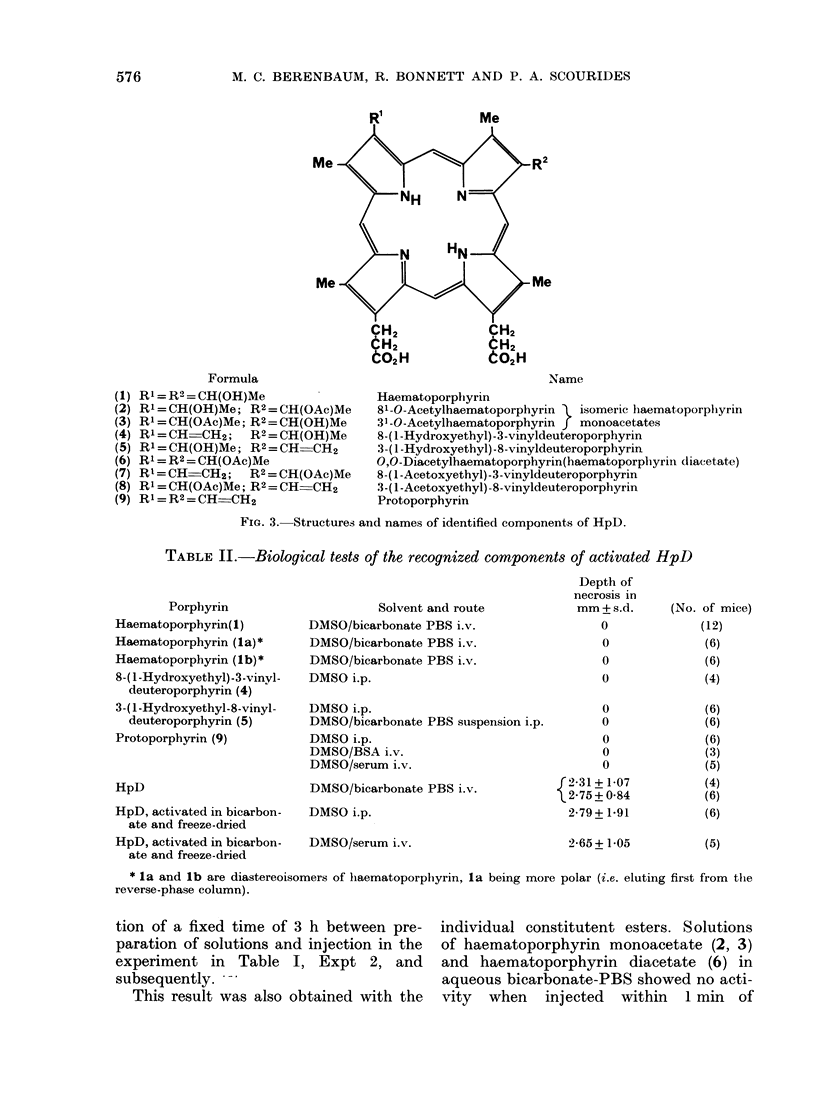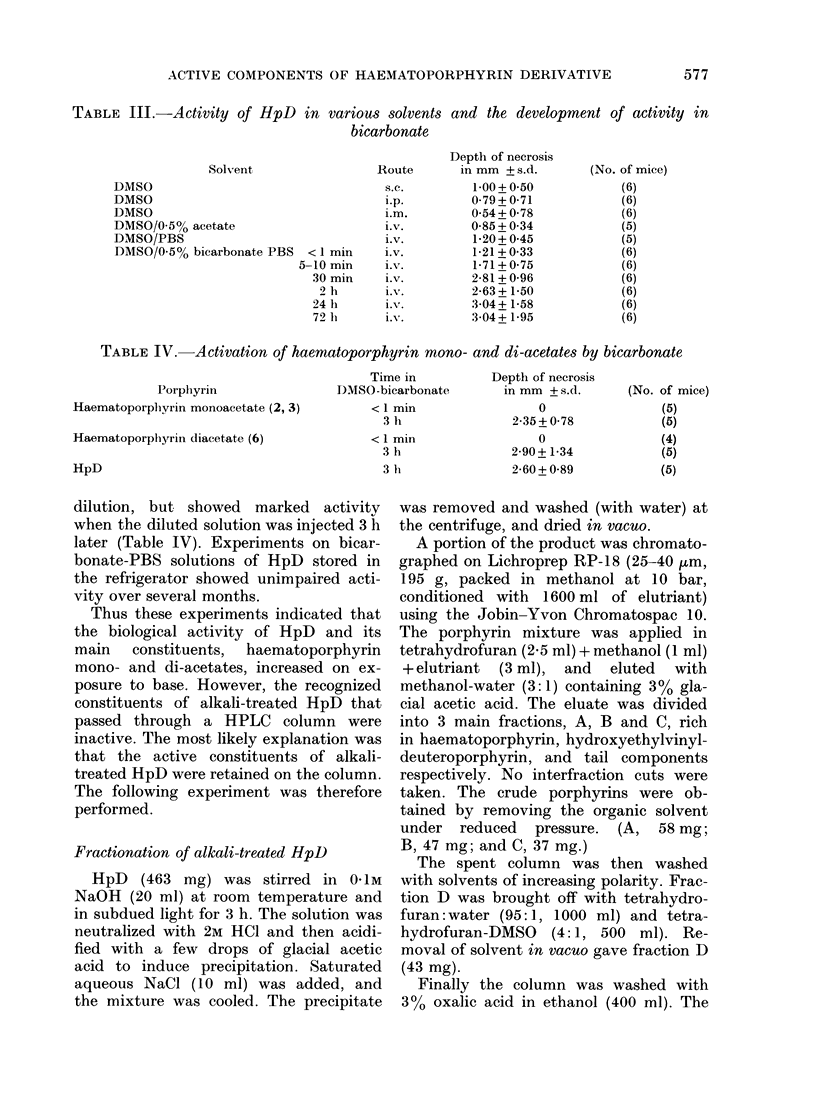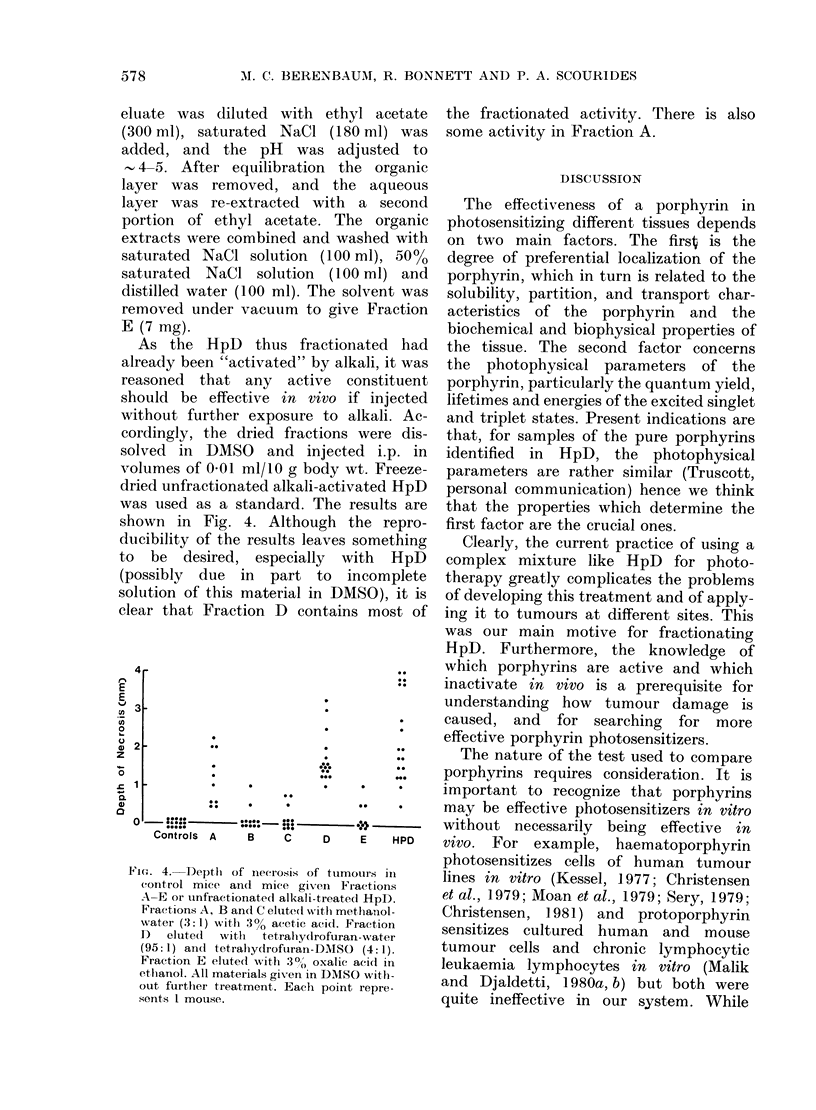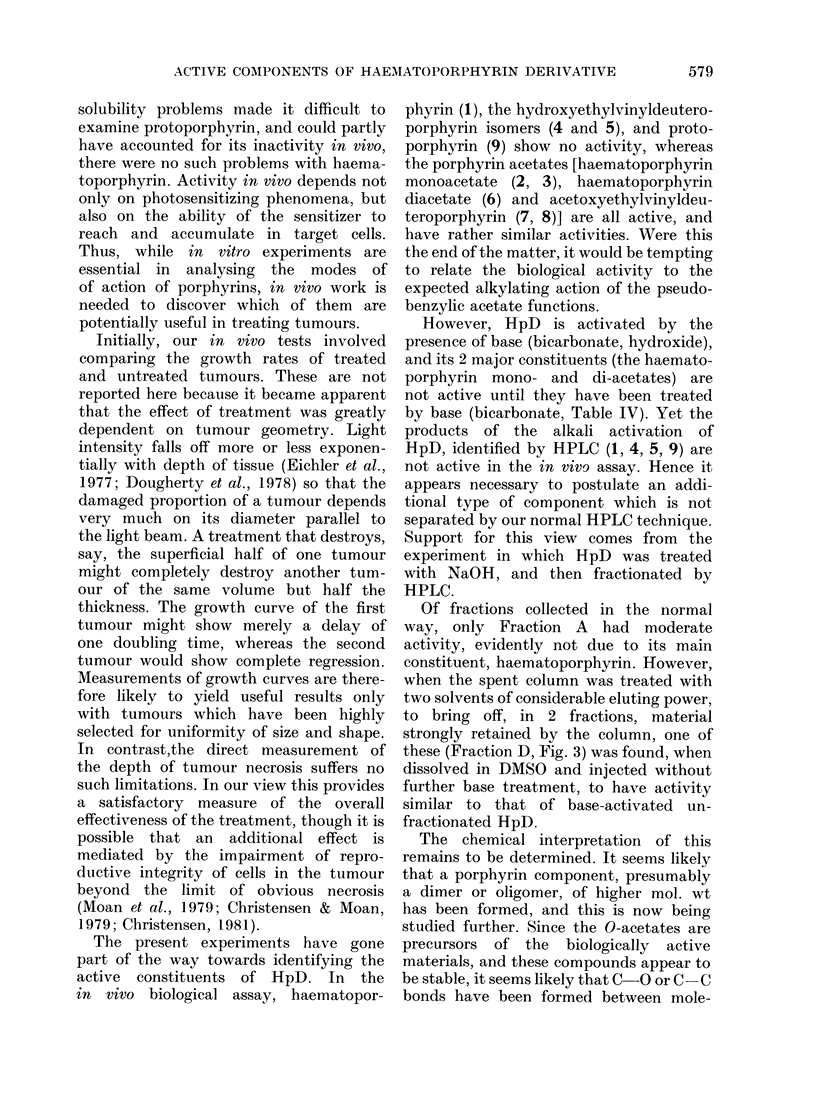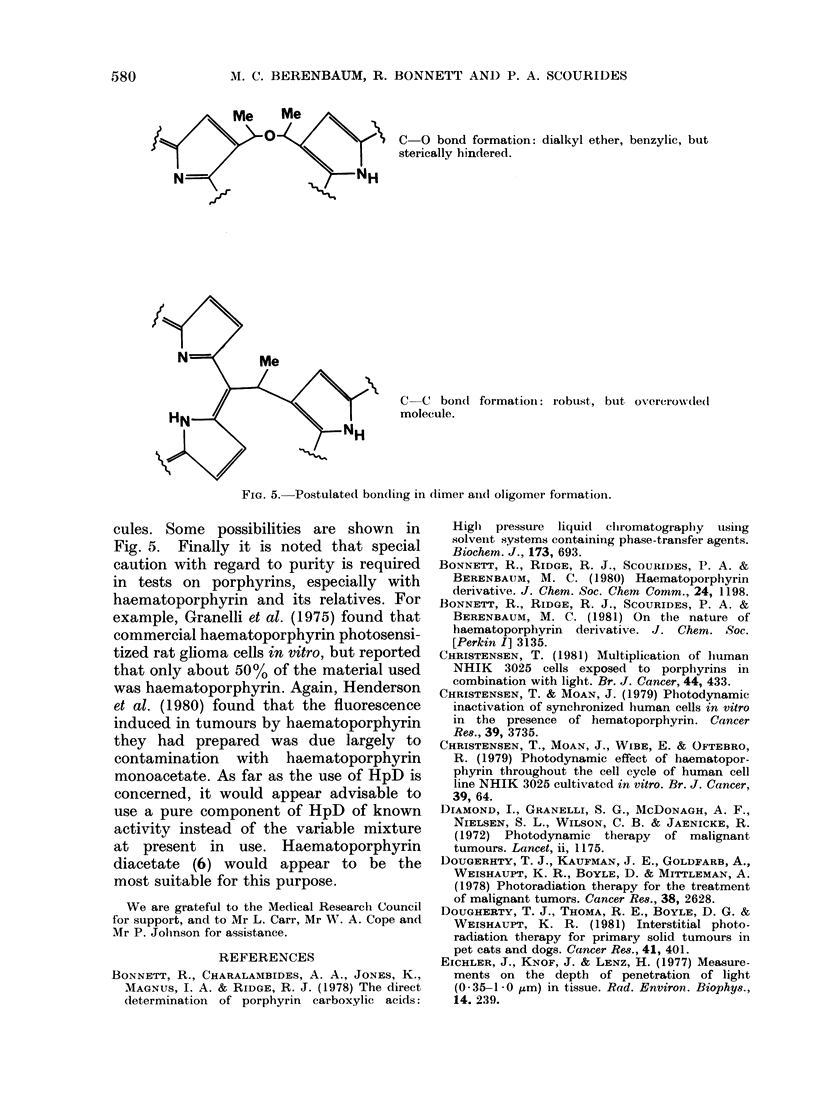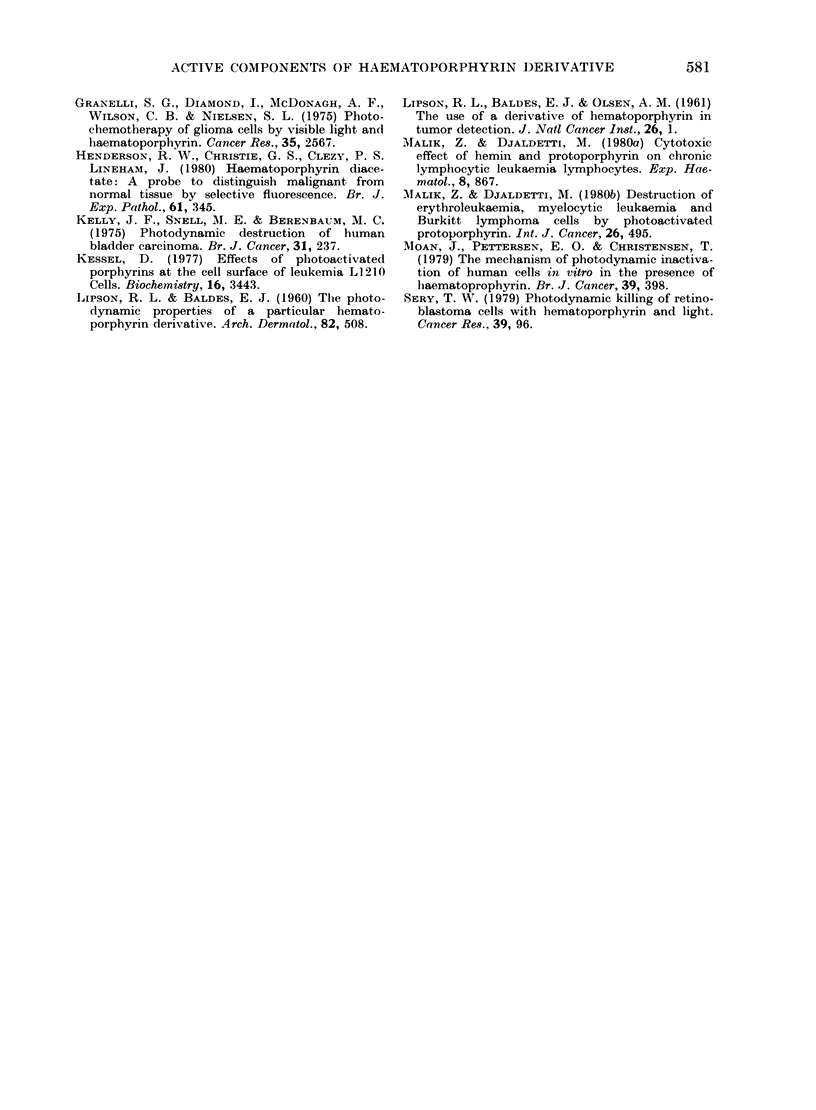# In vivo biological activity of the components of haematoporphyrin derivative.

**DOI:** 10.1038/bjc.1982.94

**Published:** 1982-04

**Authors:** M. C. Berenbaum, R. Bonnett, P. A. Scourides

## Abstract

**Images:**


Br. J. Cancer (1982) 45, 571

### *IN VIVO* BIOLOGICAL ACTIVITY OF THE COMPONENTS OF HAEMATOPORPHYRIN DERIVATIVE

M. C. BERENBAUM, R. BONNETT* AND P. A. SCOURIDES*

*From the Wellcome Laboratories of Experimental Pathology, Variety Club Research Wing, St Mary's Hospital Medical School, London W2 and the *Department of Chemistry, Queen Mary College, Mile End Road, London E1 4NS*

Received 22 September 1981 Accepted 14 December 1981

**Summary.—The *in vivo* biological activity of various fractions and components of haematoporphyrin derivative (HpD) have been determined by measuring the depth of necrosis of implanted tumours in mice exposed to light after the administration of standard doses of porphyrins dissolved in alkali.**

**In this assay, haematoporphyrin, hydroxyethylvinyldeuteroporphyrin and protoporphyrin are inactive, but the mono- and di-acetates of haematoporphyrin (which are major components of HpD) and acetoxyethylvinyldeuteroporphyrin are active.**

**However, the situation appears to be more complex than this. The normal method for preparing HpD for injection involves an alkali treatment which causes hydrolysis and elimination of the acetoxy functions, and the only recognized products (haematoporphyrin, hydroxyethylvinyldeuteroporphyrin and protoporphyrin) are inactive in the *in vivo* assay. It is concluded that the active component here is a porphyrin, possibly a dimer or oligomer, which is retained on the column during the normal separation by HPLC. This conclusion is supported by the observations that (i) the crude material obtained from the spent column is active without further alkali treatment, and (ii) activity develops over 30 min, when HpD or the mono- or di-acetates of haematoporphyrin are treated with sodium bicarbonate in aqueous DMSO.**

**The advantages of working with a pure substance (*e.g.* haematoporphyrin diacetate) rather than a mixture (HpD) are stressed.**

HAEMATOPORPHYRIN DERIVATIVE (HpD) has promise as a photosensitizing agent for the treatment of neoplasms accessible to light. On injection *in vivo*, it causes tumours exposed to intense light in the visible range to undergo rapid necrosis. The combination of HpD and light treatment has been applied with some success in the therapy of superficial tumours in man and laboratory rodents (Diamond *et al.*, 1972; Kelly *et al.*, 1975; Dougherty *et al.* 1978) and, with the aid of fibre optic light-conducting systems, in treating deep tumours in domestic animals (Dougherty *et al.*, 1981).

HpD is prepared by treating commercial haematoporphyrin dihydrochloride with sulphuric acid in glacial acetic acid (Lipson & Baldes, 1960; Lipson *et al.*, 1961). It is a complex mixture, and it is clearly important to identify the components responsible for its anti-tumour effects *in vivo* and to use these in a pure form. HpD has therefore been fractionated by preparative high-pressure liquid chromatography (Bonnett *et al.*, 1978, 1980, 1981) and we now report the activities of the various components, as judged by an *in vivo* assay using a transplantable mouse tumour.

### MATERIALS AND METHODS

*HpD and other porphyrins.*—HpD was prepared from haematoporphyrin dihydrochloride (Koch Light, Colnbrook). Full details of the preparation and fractionation using high-pressure liquid chromatography (HPLC) are given in Bonnett *et al.* (1981) which also records the preparation of authentic samples of the porphyrin carboxylic acids used here. The isomeric haematoporphyrins have not been previously characterized; they were prepared by fractionating haematoporphyrin using preparative HPLC (Scourides, unpublished).

*Preparation of porphyrin solutions.*—Because the various porphyrins differed markedly in their solubility in aqueous media and in their rates of solution, the following procedure was devised. This enabled most of them to be dissolved rapidly and conveniently. The porphyrin was dissolved in dimethyl sulphoxide (DMSO, if necessary with warming) to make a 40mg/ml solution, and this was diluted to 10 vols with 0·5% sodium bicarbonate in Dulbecco's phosphate-buffered physiological saline (PBS, Oxoid). The pH of the final solution was 7·0–7·2. In most cases an apparently stable solution was formed; the exceptions, and the methods used to deal with them, are noted below. For the studies reported in Table I, Expt 1, injections were made 0·25–3 h after making up the solution; for Expt 2 and those in Table II, a fixed time of 3 h was used.

*Mouse tumour.*—The PC6 myeloma, obtained originally from the Chester Beatty Research Institute, was passaged fortnightly in BALB/c female mice, by s.c. injection of $10^6$ viable cells. Cell viability was assessed by ability to hydrolyse fluorescein diacetate and to exclude ethidium bromide.

*Testing for biological activity.*—Tumours growing s.c. in the flanks of BALB/c female mice weighing 18–25 g were used 10–14 days after implantation, when the tumours were 6–10 mm in diameter, 5–7 mm deep, and free from evident necrosis. In general, 6 mice were used to test each fraction in each experiment, though fewer were used for the smaller fractions. The numbers of mice used in each test are specified in the Tables.

Porphyrins of known constitution were administered at a standard dose of $6{\cdot}2 \times 10^{-5}$ mol/kg. HpD, and fractions which were mixtures or of unknown constitution were assigned a mean mol. wt of 650 (*i.e.* $6{\cdot}2 \times 10^{-5}$ mol/kg$\equiv$40 mg/kg).

Solutions were injected i.v. in a volume of 0·1 ml/10 g body wt. One day later, the skin over the tumour was carefully shaved and the mouse was anaesthetized with Avertin (Winthrop Laboratories) and covered with a metal shield with a circular hole exposing the tumour. The tumour was then illuminated for 10 min with white light from a 1·6 kW xenon arc (Carl Zeiss photocoagulator) with the following filters: (a) 10 cm water (b) 2 cm 1% copper sulphate (c) 3 mm heat-absorbing filter 4602 (Optical Instrument Services) (d) Calflex B1/K1 heat-reflecting filter (Balzer's High Vacuum). Measurements with a 14BT thermopile (Laser Instrumentation) showed a delivered energy of 114 mW/cm$^2$ at the skin surface. Measurements with a thermocouple (Ellab, Copenhagen) inserted s.c. showed that the s.c. temperature did not rise more than 3–4°C during 10 min exposure to light.

A day after illumination mice were given 0·6% Evans blue in physiological saline (0·1 ml/10 g i.v.) and killed 2 h later. Tumours were fixed in formol-saline and sliced vertically to the skin surface. The depth of necrosis was measured with a stereoscopic microscope fitted with a micrometer eyepiece. Specimens were coded and examined in random order without knowledge of the treatment applied. There was generally a clear demarcation between the blue vascularized tissue and white necrotic tissue (Fig. 1) and measurement of the depth of necrosis usually presented no great difficulty if measurements were made over the centre of the tumour, where the skin surface was at right angles to the light beam. About 600 tumours were used in the work summarized here. Results from 22 of these were excluded from analysis because of interference by superficial ulceration or spontaneous necrosis (deep in the tumour and distributed irregularly without relation to the direction of illumination). Where necrosis due to illumination involved the whole tumour thickness (which occurred in about 1 tumour in 9) it was assigned a depth equal to the tumour thickness.

### RESULTS

### *Chromatographic separation*

The separation of the major components of HpD on a preparative scale by HPLC has been described (Bonnett *et al.*, 1981) and a typical separation is shown, with the components and fractions identified, in Fig. 2. Fig. 3 provides structural formulae and names. Under the preparative conditions used, the individuals of

FIG. 1.—Tumours from a control mouse (top) and 3 mice given HpD and exposed to light, showing 1, 1·5 and 2·5 mm depth of necrosis, respectively. ×4.

positionally isomeric pairs [haematoporphyrin monoacetate (**2**, **3**), hydroxyethylvinyldeuteroporphyrin (**4**, **5**), and acetoxyethylvinyldeuteroporphyrin (**7**, **8**)] were not separated, and protoporphyrin (**9**, a minor component) was not obtained as a single fraction. It is essential that the separation and work-up procedure be carried out without delay in order to minimize solvolysis and elimination of the reactive acetoxy functions.

*Biological activity of HpD fractions dissolved in alkali*

Two such separations were carried out for the purpose of biological testing. Certain minor fractions which were obtained in insufficient quantity for individual testing were combined (for a given separation) as shown in Table I, where the results for the 2 experiments are compared. The 2 experiments (*i.e.* separation plus a complete set of bioassays) were done at an interval of 6 months with 2 different batches of HpD. During this interval the focusing of the light source was improved, so that, while the mean depth of damage with a standard dose of HpD (40 mg/kg) was $1{\cdot}4 \pm 0{\cdot}6$ mm for the first set of observations, it had increased to $3{\cdot}6 \pm 0{\cdot}9$ mm by the time the second set was made. Results for tests on the HpD fractions are therefore expressed in Table I as fractions of the depth of damage concurrently observed with the standard dose of HpD.

No activity was found in the polar components (which emerged from the column first, and which were combined with other minor fractions). Haematoporphyrin and hydroxyethylvinyldeuteroporphyrin (isomers) were also inactive. On the other hand haematoporphyrin diacetate, haematoporphyrin monoacetate (isomers) and acetoxyethylvinyldeuteroporphyrin (isomers), and interfraction cuts containing these compounds were all active, and did not differ materially in activity at the doses given.

*Biological activity of known hydrolysis products of HpD (Table II)*

HpD is commonly prepared for injection by treating it with 0·1M NaOH for 1 h at room temperature (Dougherty *et al.*, 1978). Under these conditions the simple porphyrin acetates do not survive, but haematoporphyrin (**1**), hydroxyethylvinyldeuteroporphyrin (**4** and **5**), and protoporphyrin (**9**) are the major recognized constituents (Bonnett *et al.*, 1980). These compounds, prepared by standard methods, were tested in a separate series of experiments. Samples of haematoporphyrin (pure, and the commercial dihydrochloride) dissolved readily in DMSO-bicarbonate-PBS, but were found

FIG. 2.—Preparative separation of components of HpD by HPLC. The major components are identified by analytical HPLC comparisons and spectroscopic methods (Bonnett *et al.*, 1981). Because of variations in $\lambda_{max}$ with structure, peak areas do not represent relative molar amounts of the various compounds. Detector set at 400 nm.

to be inactive *in vitro*. So, too, were the individual diastereoisomers (**1a, 1b**) of haematoporphyrin. The individual isomers (**4** and **5**) of hydroxyethylvinyldeuteroporphyrin, and a sample of protoporphyrin, dissolved readily in DMSO, but precipitated on dilution with bicarbonate-PBS. Solutions in DMSO, and suspensions in DMSO-bicarbonate-PBS given i.p., were inactive. Protoporphyrin was also partially solubilized in aqueous media by mixing the solution in DMSO with 5% bovine serum albumin in PBS or with mouse serum. These preparations could be given slowly i.v. but, again, produced no detectable photosensitization of tumours (Table II).

*Effect of time on the biological activity of porphyrin acetates in aqueous bicarbonate*

In view of the effect of base on the composition of HpD, the bioassay was extended to HpD solutions in which solvolysis and elimination reactions were minimized. Three types of solution were used as follows: (i) HpD in neat DMSO, given in a volume of 0·01–0·02 ml/10 g (ii) HpD in DMSO diluted in PBS (pH 7·3) or in 0·5% sodium acetate in PBS and (iii) HpD in DMSO diluted in the usual way with bicarbonate-PBS but injected within 1 min of dilution. All these preparations showed biological activity (Table III) which was less than that observed with the bicarbonate-saline preparation administered in the normal way (when it was injected ~30 min after dilution). Timed injections showed that HpD developed biological activity in aqueous DMSO-bicarbonate-PBS solution; activity came to a maximum in ~30 min, and did not then change appreciably over 3 days (Table III). It was this observation that led to the adop-

TABLE I.—In vivo *biological activity of fractions of HpD dissolved in DMSO/bicarbonate-PBS*

| Expt 1 (294 mg HpD) | | | | Expt 2 (304 mg HpD) | | | |
|---|---|---|---|---|---|---|---|
| Fraction numbers* | Weight obtained (mg) | Identification | Activity† | Fraction numbers* | Weight obtained (mg) | Identification | Activity† |
| 1–3 }<br>9–10 } | —‡ | Polar + NaCl + interfraction cut | 0 (6) | 1–6 }<br>16–17 } | —‡ | Polar + NaCl + interfraction cut | 0 (6) |
| 4–8<br>(9–10 | 12 | Haematoporphyrin (**1**)<br>Interfraction cut) | 0 (6) | 7–9 | 11 | Haematoporphyrin (**1**) | 0 (4) |
| 11–13 | 42 | Haematoporphyrin monoacetate (**2, 3**) | 1·02 ± 0·29 (6) | 10–15 | 46 | Haematoporphyrin monoacetate (**2, 3**) | 0·66 ± 0·34 (6) |
| 14–16 | 18 | Interfraction cut | 0·22 ± 0·33 (5) | (16–17 | | Interfraction cut) | |
| 17–19 | 13 | Hydroxyethylvinyldeuteroporphyrin (**4, 5**) | 0 (6) | 18–21 | 23 | Hydroxyethylvinyldeuteroporphyrin (**4, 5**) + haematoporphyrin diacetate (**6**) | 1·08 ± 0·32 (6) |
| 20–25 | 66 | Haematoporphyrin diacetate (**6**) | 0·72 ± 0·46 (6) | 22–29 | 135 | Haematoporphyrin diacetate (**6**) | 0·78 ± 0·21 (5) |
| 26–33 | 17 | Interfraction cut | 0·47 ± 0·16 (4) | 30–38 }<br>42—46 } | 35 | Interfraction cut + minor components | 0·99 ± 0·25 (6) |
| 34 | 4 | Acetoxyethylvinyldeuteroporphyrin (**7, 8**) | 0·86 ± 0·64 (5) | 39–41 | 7 | Acetoxyethylvinyldeuteroporphyrin (**7, 8**) | 1·11 ± 0·24 (6) |
| 35–37 | 16 | Acetoxyethylvinyldeuteroporphyrin + minor components | 0·51 ± 0·38 (7) | (42–46 | | Minor components) | |

* Bonnett *et al.*, (1981).

† Depth of necrosis ± s.d. as a fraction of that obtained with HpD. (No. of animals in parentheses).

‡ Contaminated with NaCl.

| | Formula | Name |
|---|---|---|
| (1) | $R^1 = R^2 = CH(OH)Me$ | Haematoporphyrin |
| (2) | $R^1 = CH(OH)Me$; $R^2 = CH(OAc)Me$ | $8^1$-*O*-Acetylhaematoporphyrin } isomeric haematoporphyrin monoacetates |
| (3) | $R^1 = CH(OAc)Me$; $R^2 = CH(OH)Me$ | $3^1$-*O*-Acetylhaematoporphyrin } isomeric haematoporphyrin monoacetates |
| (4) | $R^1 = CH{=}CH_2$; $R^2 = CH(OH)Me$ | 8-(1-Hydroxyethyl)-3-vinyldeuteroporphyrin |
| (5) | $R^1 = CH(OH)Me$; $R^2 = CH{=}CH_2$ | 3-(1-Hydroxyethyl)-8-vinyldeuteroporphyrin |
| (6) | $R^1 = R^2 = CH(OAc)Me$ | *O,O*-Diacetylhaematoporphyrin(haematoporphyrin diacetate) |
| (7) | $R^1 = CH{=}CH_2$; $R^2 = CH(OAc)Me$ | 8-(1-Acetoxyethyl)-3-vinyldeuteroporphyrin |
| (8) | $R^1 = CH(OAc)Me$; $R^2 = CH{=}CH_2$ | 3-(1-Acetoxyethyl)-8-vinyldeuteroporphyrin |
| (9) | $R^1 = R^2 = CH{=}CH_2$ | Protoporphyrin |

FIG. 3.—Structures and names of identified components of HpD.

TABLE II.—*Biological tests of the recognized components of activated HpD*

| Porphyrin | Solvent and route | Depth of necrosis in mm ± s.d. | (No. of mice) |
|---|---|---|---|
| Haematoporphyrin(**1**) | DMSO/bicarbonate PBS i.v. | 0 | (12) |
| Haematoporphyrin (**1a**)* | DMSO/bicarbonate PBS i.v. | 0 | (6) |
| Haematoporphyrin (**1b**)* | DMSO/bicarbonate PBS i.v. | 0 | (6) |
| 8-(1-Hydroxyethyl)-3-vinyl-deuteroporphyrin (**4**) | DMSO i.p. | 0 | (4) |
| 3-(1-Hydroxyethyl-8-vinyl-deuteroporphyrin (**5**) | DMSO i.p. | 0 | (6) |
| | DMSO/bicarbonate PBS suspension i.p. | 0 | (6) |
| Protoporphyrin (**9**) | DMSO i.p. | 0 | (6) |
| | DMSO/BSA i.v. | 0 | (3) |
| | DMSO/serum i.v. | 0 | (5) |
| HpD | DMSO/bicarbonate PBS i.v. | $2{\cdot}31 \pm 1{\cdot}07$ | (4) |
| | | $2{\cdot}75 \pm 0{\cdot}84$ | (6) |
| HpD, activated in bicarbonate and freeze-dried | DMSO i.p. | $2{\cdot}79 \pm 1{\cdot}91$ | (6) |
| HpD, activated in bicarbonate and freeze-dried | DMSO/serum i.v. | $2{\cdot}65 \pm 1{\cdot}05$ | (5) |

* **1a** and **1b** are diastereoisomers of haematoporphyrin, **1a** being more polar (*i.e.* eluting first from the reverse-phase column).

tion of a fixed time of 3 h between preparation of solutions and injection in the experiment in Table I, Expt 2, and subsequently.

This result was also obtained with the individual constitutent esters. Solutions of haematoporphyrin monoacetate (**2**, **3**) and haematoporphyrin diacetate (**6**) in aqueous bicarbonate-PBS showed no activity when injected within 1 min of

TABLE III.—*Activity of HpD in various solvents and the development of activity in bicarbonate*

| Solvent | | Route | Depth of necrosis in mm ± s.d. | (No. of mice) |
|---|---|---|---|---|
| DMSO | | s.c. | 1·00 ± 0·50 | (6) |
| DMSO | | i.p. | 0·79 ± 0·71 | (6) |
| DMSO | | i.m. | 0·54 ± 0·78 | (6) |
| DMSO/0·5% acetate | | i.v. | 0·85 ± 0·34 | (5) |
| DMSO/PBS | | i.v. | 1·20 ± 0·45 | (5) |
| DMSO/0·5% bicarbonate PBS | < 1 min | i.v. | 1·21 ± 0·33 | (6) |
| | 5–10 min | i.v. | 1·71 ± 0·75 | (6) |
| | 30 min | i.v. | 2·81 ± 0·96 | (6) |
| | 2 h | i.v. | 2·63 ± 1·50 | (6) |
| | 24 h | i.v. | 3·04 ± 1·58 | (6) |
| | 72 h | i.v. | 3·04 ± 1·95 | (6) |

TABLE IV.—*Activation of haematoporphyrin mono- and di-acetates by bicarbonate*

| Porphyrin | Time in DMSO-bicarbonate | Depth of necrosis in mm ± s.d. | (No. of mice) |
|---|---|---|---|
| Haematoporphyrin monoacetate (**2**, **3**) | < 1 min | 0 | (5) |
| | 3 h | 2·35 ± 0·78 | (5) |
| Haematoporphyrin diacetate (**6**) | < 1 min | 0 | (4) |
| | 3 h | 2·90 ± 1·34 | (5) |
| HpD | 3 h | 2·60 ± 0·89 | (5) |

dilution, but showed marked activity when the diluted solution was injected 3 h later (Table IV). Experiments on bicarbonate-PBS solutions of HpD stored in the refrigerator showed unimpaired activity over several months.

Thus these experiments indicated that the biological activity of HpD and its main constituents, haematoporphyrin mono- and di-acetates, increased on exposure to base. However, the recognized constituents of alkali-treated HpD that passed through a HPLC column were inactive. The most likely explanation was that the active constituents of alkali-treated HpD were retained on the column. The following experiment was therefore performed.

### *Fractionation of alkali-treated HpD*

HpD (463 mg) was stirred in 0·1M NaOH (20 ml) at room temperature and in subdued light for 3 h. The solution was neutralized with 2M HCl and then acidified with a few drops of glacial acetic acid to induce precipitation. Saturated aqueous NaCl (10 ml) was added, and the mixture was cooled. The precipitate was removed and washed (with water) at the centrifuge, and dried *in vacuo*.

A portion of the product was chromatographed on Lichroprep RP-18 (25–40 μm, 195 g, packed in methanol at 10 bar, conditioned with 1600 ml of elutriant) using the Jobin–Yvon Chromatospac 10. The porphyrin mixture was applied in tetrahydrofuran (2·5 ml) + methanol (1 ml) + elutriant (3 ml), and eluted with methanol-water (3:1) containing 3% glacial acetic acid. The eluate was divided into 3 main fractions, A, B and C, rich in haematoporphyrin, hydroxyethylvinyldeuteroporphyrin, and tail components respectively. No interfraction cuts were taken. The crude porphyrins were obtained by removing the organic solvent under reduced pressure. (A, 58 mg; B, 47 mg; and C, 37 mg.)

The spent column was then washed with solvents of increasing polarity. Fraction D was brought off with tetrahydrofuran:water (95:1, 1000 ml) and tetrahydrofuran-DMSO (4:1, 500 ml). Removal of solvent *in vacuo* gave fraction D (43 mg).

Finally the column was washed with 3% oxalic acid in ethanol (400 ml). The eluate was diluted with ethyl acetate (300 ml), saturated NaCl (180 ml) was added, and the pH was adjusted to ~4–5. After equilibration the organic layer was removed, and the aqueous layer was re-extracted with a second portion of ethyl acetate. The organic extracts were combined and washed with saturated NaCl solution (100 ml), 50% saturated NaCl solution (100 ml) and distilled water (100 ml). The solvent was removed under vacuum to give Fraction E (7 mg).

As the HpD thus fractionated had already been "activated" by alkali, it was reasoned that any active constituent should be effective *in vivo* if injected without further exposure to alkali. Accordingly, the dried fractions were dissolved in DMSO and injected i.p. in volumes of 0·01 ml/10 g body wt. Freeze-dried unfractionated alkali-activated HpD was used as a standard. The results are shown in Fig. 4. Although the reproducibility of the results leaves something to be desired, especially with HpD (possibly due in part to incomplete solution of this material in DMSO), it is clear that Fraction D contains most of the fractionated activity. There is also some activity in Fraction A.

FIG. 4.—Depth of necrosis of tumours in control mice and mice given Fractions A–E or unfractionated alkali-treated HpD. Fractions A, B and C eluted with methanol-water (3:1) with 3% acetic acid. Fraction D eluted with tetrahydrofuran-water (95:1) and tetrahydrofuran-DMSO (4:1). Fraction E eluted with 3% oxalic acid in ethanol. All materials given in DMSO without further treatment. Each point represents 1 mouse.

### DISCUSSION

The effectiveness of a porphyrin in photosensitizing different tissues depends on two main factors. The first is the degree of preferential localization of the porphyrin, which in turn is related to the solubility, partition, and transport characteristics of the porphyrin and the biochemical and biophysical properties of the tissue. The second factor concerns the photophysical parameters of the porphyrin, particularly the quantum yield, lifetimes and energies of the excited singlet and triplet states. Present indications are that, for samples of the pure porphyrins identified in HpD, the photophysical parameters are rather similar (Truscott, personal communication) hence we think that the properties which determine the first factor are the crucial ones.

Clearly, the current practice of using a complex mixture like HpD for phototherapy greatly complicates the problems of developing this treatment and of applying it to tumours at different sites. This was our main motive for fractionating HpD. Furthermore, the knowledge of which porphyrins are active and which inactivate *in vivo* is a prerequisite for understanding how tumour damage is caused, and for searching for more effective porphyrin photosensitizers.

The nature of the test used to compare porphyrins requires consideration. It is important to recognize that porphyrins may be effective photosensitizers *in vitro* without necessarily being effective *in vivo*. For example, haematoporphyrin photosensitizes cells of human tumour lines *in vitro* (Kessel, 1977; Christensen *et al.*, 1979; Moan *et al.*, 1979; Sery, 1979; Christensen, 1981) and protoporphyrin sensitizes cultured human and mouse tumour cells and chronic lymphocytic leukaemia lymphocytes *in vitro* (Malik and Djaldetti, 1980*a*, *b*) but both were quite ineffective in our system. While solubility problems made it difficult to examine protoporphyrin, and could partly have accounted for its inactivity *in vivo*, there were no such problems with haematoporphyrin. Activity *in vivo* depends not only on photosensitizing phenomena, but also on the ability of the sensitizer to reach and accumulate in target cells. Thus, while *in vitro* experiments are essential in analysing the modes of of action of porphyrins, *in vivo* work is needed to discover which of them are potentially useful in treating tumours.

Initially, our *in vivo* tests involved comparing the growth rates of treated and untreated tumours. These are not reported here because it became apparent that the effect of treatment was greatly dependent on tumour geometry. Light intensity falls off more or less exponentially with depth of tissue (Eichler *et al.*, 1977; Dougherty *et al.*, 1978) so that the damaged proportion of a tumour depends very much on its diameter parallel to the light beam. A treatment that destroys, say, the superficial half of one tumour might completely destroy another tumour of the same volume but half the thickness. The growth curve of the first tumour might show merely a delay of one doubling time, whereas the second tumour would show complete regression. Measurements of growth curves are therefore likely to yield useful results only with tumours which have been highly selected for uniformity of size and shape. In contrast,the direct measurement of the depth of tumour necrosis suffers no such limitations. In our view this provides a satisfactory measure of the overall effectiveness of the treatment, though it is possible that an additional effect is mediated by the impairment of reproductive integrity of cells in the tumour beyond the limit of obvious necrosis (Moan *et al.*, 1979; Christensen & Moan, 1979; Christensen, 1981).

The present experiments have gone part of the way towards identifying the active constituents of HpD. In the *in vivo* biological assay, haematoporphyrin (**1**), the hydroxyethylvinyldeuteroporphyrin isomers (**4** and **5**), and protoporphyrin (**9**) show no activity, whereas the porphyrin acetates [haematoporphyrin monoacetate (**2**, **3**), haematoporphyrin diacetate (**6**) and acetoxyethylvinyldeuteroporphyrin (**7**, **8**)] are all active, and have rather similar activities. Were this the end of the matter, it would be tempting to relate the biological activity to the expected alkylating action of the pseudobenzylic acetate functions.

However, HpD is activated by the presence of base (bicarbonate, hydroxide), and its 2 major constituents (the haematoporphyrin mono- and di-acetates) are not active until they have been treated by base (bicarbonate, Table IV). Yet the products of the alkali activation of HpD, identified by HPLC (**1**, **4**, **5**, **9**) are not active in the *in vivo* assay. Hence it appears necessary to postulate an additional type of component which is not separated by our normal HPLC technique. Support for this view comes from the experiment in which HpD was treated with NaOH, and then fractionated by HPLC.

Of fractions collected in the normal way, only Fraction A had moderate activity, evidently not due to its main constituent, haematoporphyrin. However, when the spent column was treated with two solvents of considerable eluting power, to bring off, in 2 fractions, material strongly retained by the column, one of these (Fraction D, Fig. 3) was found, when dissolved in DMSO and injected without further base treatment, to have activity similar to that of base-activated unfractionated HpD.

The chemical interpretation of this remains to be determined. It seems likely that a porphyrin component, presumably a dimer or oligomer, of higher mol. wt has been formed, and this is now being studied further. Since the *O*-acetates are precursors of the biologically active materials, and these compounds appear to be stable, it seems likely that C—O or C—C bonds have been formed between mole-

C—O bond formation: dialkyl ether, benzylic, but sterically hindered.

C—C bond formation: robust, but overcrowded molecule.

FIG. 5.—Postulated bonding in dimer and oligomer formation.

cules. Some possibilities are shown in Fig. 5. Finally it is noted that special caution with regard to purity is required in tests on porphyrins, especially with haematoporphyrin and its relatives. For example, Granelli *et al.* (1975) found that commercial haematoporphyrin photosensitized rat glioma cells *in vitro*, but reported that only about 50% of the material used was haematoporphyrin. Again, Henderson *et al.* (1980) found that the fluorescence induced in tumours by haematoporphyrin they had prepared was due largely to contamination with haematoporphyrin monoacetate. As far as the use of HpD is concerned, it would appear advisable to use a pure component of HpD of known activity instead of the variable mixture at present in use. Haematoporphyrin diacetate (**6**) would appear to be the most suitable for this purpose.

We are grateful to the Medical Research Council for support, and to Mr L. Carr, Mr W. A. Cope and Mr P. Johnson for assistance.

REFERENCES

BONNETT, R., CHARALAMBIDES, A. A., JONES, K., MAGNUS, I. A. & RIDGE, R. J. (1978) The direct determination of porphyrin carboxylic acids: High pressure liquid chromatography using solvent systems containing phase-transfer agents. *Biochem. J.*, **173**, 693.

BONNETT, R., RIDGE, R. J., SCOURIDES, P. A. & BERENBAUM, M. C. (1980) Haematoporphyrin derivative. *J. Chem. Soc. Chem Comm.*, **24**, 1198.

BONNETT, R., RIDGE, R. J., SCOURIDES, P. A. & BERENBAUM, M. C. (1981) On the nature of haematoporphyrin derivative. *J. Chem. Soc.* [*Perkin I*] 3135.

CHRISTENSEN, T. (1981) Multiplication of human NHIK 3025 cells exposed to porphyrins in combination with light. *Br. J. Cancer*, **44**, 433.

CHRISTENSEN, T. & MOAN, J. (1979) Photodynamic inactivation of synchronized human cells *in vitro* in the presence of hematoporphyrin. *Cancer Res.*, **39**, 3735.

CHRISTENSEN, T., MOAN, J., WIBE, E. & OFTEBRO, R. (1979) Photodynamic effect of haematoporphyrin throughout the cell cycle of human cell line NHIK 3025 cultivated *in vitro*. *Br. J. Cancer*, **39**, 64.

DIAMOND, I., GRANELLI, S. G., MCDONAGH, A. F., NIELSEN, S. L., WILSON, C. B. & JAENICKE, R. (1972) Photodynamic therapy of malignant tumours. *Lancet*, ii, 1175.

DOUGERHTY, T. J., KAUFMAN, J. E., GOLDFARB, A., WEISHAUPT, K. R., BOYLE, D. & MITTLEMAN, A. (1978) Photoradiation therapy for the treatment of malignant tumors. *Cancer Res.*, **38**, 2628.

DOUGHERTY, T. J., THOMA, R. E., BOYLE, D. G. & WEISHAUPT, K. R. (1981) Interstitial photoradiation therapy for primary solid tumours in pet cats and dogs. *Cancer Res.*, **41**, 401.

EICHLER, J., KNOF, J. & LENZ, H. (1977) Measurements on the depth of penetration of light (0·35–1·0 μm) in tissue. *Rad. Environ. Biophys.*, **14**, 239.

GRANELLI, S. G., DIAMOND, I., McDONAGH, A. F., WILSON, C. B. & NIELSEN, S. L. (1975) Photochemotherapy of glioma cells by visible light and haematoporphyrin. *Cancer Res.*, **35**, 2567.

HENDERSON, R. W., CHRISTIE, G. S., CLEZY, P. S. LINEHAM, J. (1980) Haematoporphyrin diacetate: A probe to distinguish malignant from normal tissue by selective fluorescence. *Br. J. Exp. Pathol.*, **61**, 345.

KELLY, J. F., SNELL, M. E. & BERENBAUM, M. C. (1975) Photodynamic destruction of human bladder carcinoma. *Br. J. Cancer*, **31**, 237.

KESSEL, D. (1977) Effects of photoactivated porphyrins at the cell surface of leukemia L1210 Cells. *Biochemistry*, **16**, 3443.

LIPSON, R. L. & BALDES, E. J. (1960) The photodynamic properties of a particular hematoporphyrin derivative. *Arch. Dermatol.*, **82**, 508.

LIPSON, R. L., BALDES, E. J. & OLSEN, A. M. (1961) The use of a derivative of hematoporphyrin in tumor detection. *J. Natl Cancer Inst.*, **26**, 1.

MALIK, Z. & DJALDETTI, M. (1980*a*) Cytotoxic effect of hemin and protoporphyrin on chronic lymphocytic leukaemia lymphocytes. *Exp. Haematol.*, **8**, 867.

MALIK, Z. & DJALDETTI, M. (1980*b*) Destruction of erythroleukaemia, myelocytic leukaemia and Burkitt lymphoma cells by photoactivated protoporphyrin. *Int. J. Cancer*, **26**, 495.

MOAN, J., PETTERSEN, E. O. & CHRISTENSEN, T. (1979) The mechanism of photodynamic inactivation of human cells *in vitro* in the presence of haematoprophyrin. *Br. J. Cancer*, **39**, 398.

SERY, T. W. (1979) Photodynamic killing of retinoblastoma cells with hematoporphyrin and light. *Cancer Res.*, **39**, 96.